# Scoring System to Personalize Management of Emphysematous Pyelonephritis

**DOI:** 10.5152/tud.2024.23165

**Published:** 2024-05-01

**Authors:** Ankur Mittal, Deepak Kumar, Vikas Kumar Panwar, Rohit Ranjan, Shiv Charan Navriya, Akshay Upadhyaya, Harshit Agarwal, Gautam Shubhankar, Arup Kumar Mandal

**Affiliations:** Department of Urology, All India Institute of Medical Sciences, Rishikesh, Uttarakhand, India

**Keywords:** Pyelonephritis, prognosis, mortality, risk factors, treatment outcome

## Abstract

**Objective:**

Emphysematous pyelonephritis (EPN) is a life-threatening condition that requires prompt diagnosis and treatment. The prognosis of EPN is variable, and there is no single treatment that is universally effective.

**Materials and Methods:**

In this study, we developed a scoring system to predict the prognosis of EPN and to guide management. The scoring system was developed based on a retrospective analysis of 91 patients with EPN. Nineteen risk factors for emphysematous pyelonephritis were assessed with univariate and multivariate analysis.

**Results:**

Seven factors were found significant on analysis. The scoring system was developed by including these 7 risk factors: renal stone disease, leukocytosis, raised creatinine, EPN grade, and septic shock. The score ranged from 1 to 18, with a higher score indicating a worse prognosis. The scoring system was able to stratify patients into three risk groups: good risk, intermediate risk, and poor risk. The scoring system can be used to personalize the management of EPN. Patients in the good-risk group may be managed with conservative treatment, while patients in the intermediate-risk and poor-risk groups may require intervention, such as DJ stenting, percutaneous nephrolithotomy or nephrectomy. The scoring system is a valuable tool for predicting the prognosis of EPN and guiding management. It can help clinicians to tailor treatment to the individual patient and to improve outcomes.

**Conclusion:**

The prognostic score helps identify patients who are at high risk. This score helps in the selection of appropriate management options.

**Main Points**

- Emphysematous pyelonephritis (EPN) is a severe kidney infection with high mortality rates.
- Diabetes mellitus, nephrolithiasis, and septic shock are common risk factors.
- A new scoring system is proposed to predict prognosis based on these factors.
  The study analyzed three types of interventions: conservative management, DJ stent placement, and PCN placement.
  The success of each intervention varies depending on the patient’s risk group.
- The developed scoring system effectively stratifies patients into good, intermediate, and poor risk groups.

## Introduction

Emphysematous pyelonephritis (EPN) is an acute necrotizing infection of the kidney.^1^ The first case of emphysematous pyelonephritis was reported by Kelly and MacCallum in 1898, and the term “emphysematous pyelonephritis” was coined by Schultz and Klorfein.^2,3^

Diabetes mellitus (DM) is the most common underlying etiology. More than 90% of cases of EPN occur in patients with DM.^4,5^ Other causes include obstruction in the kidney or ureter either due to a stone or other cause, immunocompromised status, drug abuse, alcoholism, neurogenic bladder, and anatomically abnormal bladder.^6-8^

The most common organism associated with this is Escherichia coli, and other causative organisms include Proteus mirabilis, Klebsiella pneumoniae, Group D Streptococcus, and coagulase-negative Staphylococcus.^9^

Mortality due to EPN ranges between 11% and 42%, as reported in a recent meta-analysis.^10^ Several risk factors have been identified in the literature to be associated with increased mortality.^11-14^

In the case of emphysematous pyelonephritis, the management choice is crucial. The likelihood of fatality can rise if an improper intervention is chosen.

Here, we reviewed retrospectively the patients with emphysematous pyelonephritis to identify risk factors affecting the outcome of EPN and, we developed a scoring system to aid in the customization of management.

## Material and Methods

We have done a retrospective analysis of patients with emphysematous pyelonephritis who were managed at our center between June 2017 and February 2023. Informed consent was taken from all the participants. Ethical clearance was taken from the institutional ethical committee. (All India Institute of Medical Sciences/IEC/20/299)

### Inclusion Criteria

1. Patients with emphysematous pyelonephritis who were managed at our center

### Exclusion Criteria

1. Patients in whom the initial intervention was done outside.
2. Patients, whose records were not available.

Demographic, clinical, biochemical, and management data were obtained and evaluated. Multiple factors are mentioned in the literature to be associated with poor prognosis. We analyzed 19 risk factors during this study. Risk factors were selected based on previous studies.^11-14^ Prognosis was defined based on mortality and morbidity. Good and poor prognoses were defined as mentioned below:

1. Poor prognosis − prolonged hospital stay (>10 days) or mortality was considered poor outcomes during our study.
2. Good prognosis − a hospital stay equal to or less than 10 days and discharge in stable condition was considered a good outcome.

Univariate analysis and multivariate analysis were used on all risk factors to assess their effect on an outcome. Risk factors that were found significant in univariate analysis were again analyzed with multivariate analysis.

A prognostic score was developed based on risk factors which were found significant in our analysis. Good, intermediate, and poor prognostic risk groups were constructed based on prognostic score.

All the patients were managed with either antibiotics alone or with antibiotics plus DJ stent placement or with antibiotics plus percutaneous nephrostomy (PCN) placement. Percutaneous drain (PCD) was placed in all patients who had a 3 cm or more collection in the perinephric or pararenal space.

Based on this management, patients were divided into three categories:

Type 1 intervention: patients who were managed conservatively with antibiotics

Type 2 intervention: patients who were managed with DJ stent placement +/– PCD placement

Type 3 intervention: patients who were managed with PCN placement +/– PCD placement.

In our study, no patient underwent an upfront nephrectomy without a previous intervention.

The outcome of treatment was defined in terms of success and failure.

Failure – need for further intervention or mortality.

Success – no need for further intervention and no mortality.

The outcome of treatment for each category of intervention was analyzed according to good, intermediate, and poor prognostic risk groups. Final analysis was done to assess the best treatment modality for each prognostic group.

### Statistical Analysis

All the data were tabulated in Microsoft Excel for Windows and analyzed using Statistical Package for the Social Sciences (SPSS) version 26.0 (IBM SPSS Corp., Armonk, NY, USA). Because of the retrospective nature of the study, no sample size calculation was done upfront. The normalcy of the distribution of continuous variables was tested using the one-sample Kolmogorov-Smirnov test. All continuous variables were found to have a non-normal distribution; therefore, continuous variables were presented in the median (interquartile range). Discrete data and categorical variables were presented in means and percentages. The Mann−Whitney U-test and Kruskal Wallis test were applied for comparing 2 or more independent samples, respectively. A forward likelihood ratio binary logistic regression analysis was performed to compute independent risk factors predicting the outcome of emphysematous pyelonephritis. The predictive value of the scoring system was evaluated using receiver operating characteristic curve (ROC) analysis.

## Results

A total of 91 patients were analyzed during the study. Table 1 shows all the variables in the study. The mean age of patients with emphysematous pyelonephritis was 52.88 +/– 13.69 years. About 61.53% of patients were females, and 38.45% were males. About 68.1% of patients were diabetic, and 33% of patients had shock (MAP [Mean arterial pressure] < 65) at the time of presentation. 83.5% of patients had SIRS and 5.5% of patients had multiple organ dysfunction syndrome (MODS) at the time of presentation. About 59.3% of patients had associated renal stone disease.

Laboratory data showed the average hemoglobin level was 10 (4) [median (interquartile range)], and 41.8% of patients had moderate to severe anemia at the time of presentation. The average total leukocyte count (TLC) count was 14 000/mm^3^ (16 000), and 54.9% of patients had leukocytosis. The average platelet count was 134 k/mm^3^ (135 k), and 60.4% of patients had thrombocytopenia at the time of presentation. The average creatinine value was 1.9 (2.1) mg/dL, and 53.8% of patients had raised creatinine (>1.5 mg/dL). Seventy-eight patients had positive urine cultures and 22% of patients had no growth on urine culture.

Management data showed 15.3% of patients were managed conservatively, and 41.8% and 42.9% of patients were managed with DJ stent and PCN placement, respectively. Percutaneous drain insertion was done in 33% of patients.

The mortality rate was 8.8%, and a total of 8 patients expired during hospital admission.

Univariate analysis was done to see the effect of variables on the outcome of emphysematous pyelonephritis. Nineteen variables were analyzed for their association with prognosis. Out of 19, 7 factors were found significant in the univariate analysis, and 3 factors were found significant in multivariate analysis as shown in Table 2 and Table 3. Each factor assigned a score based on the odds ratio.

A prognostic score was developed based on significant variables (Table 4). The minimum score was 1, and the maximum score was 18. Three risk groups were constructed based on prognostic scores, including good risk (1-6), intermediate risk (7-12), and poor risk group (13-18).

**Figure 1.**
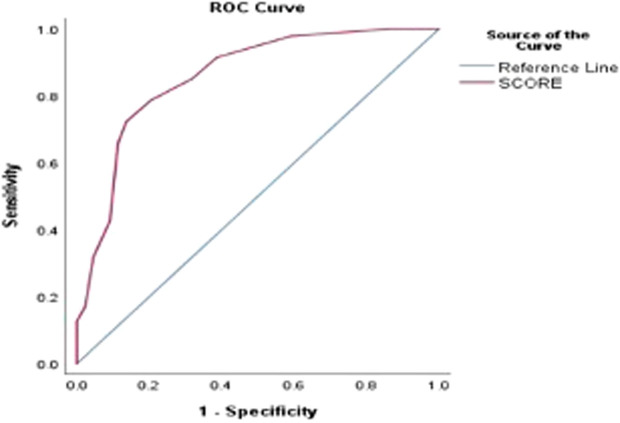
ROC curve for prognostic score.

The ROC curve analysis was done individually for all risk factors. A ROC curve analysis for the prognostic score was also done, as shown in Figure 1. The area under the curve for the prognostic score was 0.859, which was significantly higher than individual risk factor.

Outcome of all treatment modalities was compared for each risk group as shown in Table 5.

The outcome of each intervention was analyzed for all 3 prognostic risk groups.

## Discussion

Emphysematous pyelonephritis is a severe necrotizing infection mostly seen in diabetic patients. Up to 90% of EPN patients can have DM.^15^ In our study, 62.1% of patients had DM. Diabetic patients had higher chances of urinary tract infections in comparison to the nondiabetic population.^15^ In our study, we found a female-to-male ratio of 1.6 : 1. Females have higher probability of urinary tract infections.^16^ Ascending infection can increase the risk of emphysematous pyelonephritis in females.

A recent systematic review has suggested an association of nephrolithiasis with emphysematous pyelonephritis in 22% of patients, and Sanford et al found 57% of patients had nephrolithiasis in patients with emphysematous pyelonephritis.^17,18^ In our study, we found that 59.3% of patients had nephrolithiasis. Literature has suggested 20-30% of EPN patients had septic shock at the time of presentation. In our study, we found that 33% of patients with EPN presented with shock.^19^ We found a positive urine culture in 78% of patients. Literature has suggested 45-90% of patients have a positive urine culture and* E. coli* was the most common.^20,21^

Multiple studies have been done to analyze the prognostic factors of emphysematous pyelonephritis.^11-14,22,23^ In a meta-analysis by Falaga et al, hypotension, disturbances in consciousness, raised serum creatinine, and thrombocytopenia were associated with increased mortality.^10^ Bilateral EPN and type I EPN, according to Wen et al’s classification, were also associated with increased mortality. Kapoor et al^12^ found the renal failure, thrombocytopenia, altered sensorium and severe hyponatremia as the significant risk factor for the mortality in EPN patients. A study by Olvera-Posada et al^13^ showed the higher mortality in patients having raised total leukocyte count, multiorgan failure, altered sensorium and hyperglycaemia.

Based on previous studies, we have evaluated nineteen factors for their effect on the prognosis of EPN. We found seven factors significant in univariate analysis (renal stone, leucocytosis, raised creatinine, SIRS, septic shock, urine culture, and EPN grade) and three factors significant in multivariate analysis. We constructed a prognostic score based on these risk factors. ROC analysis was done for the prognostic score. The area under the curve was 0.827, which suggests the good predictive value of the prognostic score.

According to prognostic risk groups, we had 19 (20.9%) patients in the good risk group, 47 (51.6%) patients in the intermediate risk group, and 25 (27.5%) patients in the poor risk group.

Fourteen (15.4%) patients were managed with type 1 intervention (conservative management including antibiotics and diabetes control), 38 (41.8%) patients were managed with type 2 intervention (DJ stent +/− PCD placement), and 39 (42.9%) patients were managed with type 3 intervention (PCN +/− PCD placement).

The success rate of good-risk group patients was 80% with type 1 intervention, 90.9% with type 2 intervention, and 100% with type 3 intervention. In the good risk group, there was no significant difference in type 1, type 2, and type 3 intervention groups.

The success rate of intermediate-risk group patients was 25% with type 1 intervention, 60% with type 2 intervention, and 94.7% with type 3 intervention. In the intermediate group, the success rate of the type 1 group was significantly lower than type 2 and type 3.

The success rate of poor-risk group patients was 25% with type 2 intervention and 58.8% with type 3 intervention. No patient in the poor-risk group was managed with type 1 intervention. In the poor-risk group, the success rate of the type 2 group was significantly lower than the type 3 intervention.

Emphysematous pyelonephritis is a severe necrotizing infection of the kidney and is associated with high mortality. The type of intervention is not standardized for emphysematous pyelonephritis patients. The prognostic score helps identify patients who are at high risk. This score helps in the selection of appropriate management options.

## Limitations

The first limitation of our study is a retrospective analysis. Second, we have analyzed the use of the scoring system for the selection of interventions according to the risk group. But external validation of our scoring system is needed. Many other risk factors (CRP, procalcitonin, and cardiac reserves) which could be important for the prognosis were not included in the study because of unavailability of the data due to retrospective analysis.

## Figures and Tables

**Table 1.** Demographic, Clinicopathological, Laboratory, and Management Data of Patients

Variables	
Age (mean ± standard deviation) in years	52.88 ± 13.69
Female/male	56/35
Female/male ratio	1.6/1
Duration of symptoms before presentation (mean ± SD) in days	3.92 ± 1.62
Symptoms (In percentage) Fever Pain abdomen Nausea and vomiting	85 (93.4%) 80 (89%) 41 (45.1%)
Diabetes mellitus (in percentage)	62 (68.1%)
Renal stone disease (in percentage)	54 (59.3%)
Shock at time of presentation	30 (33%)
SIRS	76 (83.5%)
MODS	5 (5.5%)
Hemoglobin g/dL (median (iqr))	10 (4)
Anemia <9 g/dL	39 (42.85%)
TLC count	14 000/mm^3^(16 000)
Leukocytosis	49 (53.8%)
Platelet count	139k (123k)
Thrombocytopenia	53 (58.2%)
Creatinine level	1.8 (2.0)
Raised creatinine	49 (53.8%)
Albumin level	3.1 (0.8)
Hypoalbuminemia	34 (37.4%)
Positive urine culture	71 (78%)
Management Conservative DJS stent PCN placement	14 (15.4%) 38 (41.8%) 39 (42.9%)
PCD placement	30 (33%)
Mortality	8 (8.8%)

MODS, multiple organ dysfunction syndrome; PCD, percutaneous drain; TLC, total leukocyte count; PCN, percutaneous nephrostomy; SIRS, systematic inflammatory response syndrome.

**Table 2.** Univariate Analysis of Variables with Outcome of Emphysematous Pyelonephritis

Variables	Odds Ratio	*P*
Age	0.97(0.94-1.002)	.64
Gender	1.107(0.468-2.619)	.817
Duration of symptoms	0.908(0.7-1.178)	.468
Diabetes mellitus	1.495(0.616-3.626)	.374
Renal stones	2.582(1.092-6.105)	.031
Anemia	1.066(0.746-1.522)	.72
Leukocytosis	4.577(1.886-11.106)	.001
Thrombocytopenia	0.428(0.183-1.003)	.051
Raised creatinine Value (>1.5 mg/dL)	3.391(1.433-8.020)	.005
Sodium	0.930(0.587-1.008)	.078
Potassium	0.885(0.571-1.374)	.587
EPN grade	29.091(3.332-253.964)	.000
EPN side	1.635(0.365-7.326)	.701
Hypoalbuminemia	0.426(0.177-1.024)	.056
SIRS	3.583(1.046-12.272)	.042
Septic shock	7.197(2.559-20.224)	.000
MODS	4.000(0.429-37.263)	.223
Altered sensorium	4.000(0.429-37.263)	.223
Urine culture	9.000(0.854-94.899)	.020

MODS, multiple organ dysfunction syndrome; SIRS, systematic inflammatory response syndrome.

**Table 3.** Multivariate Analysis of Variables Which Were Significant in Univariate Analysis

Variables	Odds Ratio	*P*	Score Attributed
Renal stones	6.073(1.710-21.569)	.005	3
Leukocytosis	6.090(1.824-20.334)	.003	3
Septic shock	5.742(1.279-25.774)	.023	3
Raised Creatinine Value(>1.5 mg/dL)	3.179(0.978-10.341)	.048	2
Urine culture	0.337(0.083-1.376)	.130	1
SIRS	1.052(0.220-5.044)	.949	1
EPN crade	–	.506	1-5

EPN, emphysematous pyelonephritis; SIRS, systematic inflammatory response syndrome.

**Table 4.** Prognostic Score System (Minimum Value: 1 and Maximum Value: 18)

Variables	Score					
	0	1	2	3	4	5
Renal stone disease	Absent			Present		
Septic shock	Absent			Present		
Leukocytosis	Absent			Present		
Creatinine raised (>1.5)	Absent		Present			
SIRS	Absent	Present				
Positive urine culture	Absent	Present				
EPN grade	–	1	2	3a	3b	4

EPN, emphysematous pyelonephritis; SIRS, systematic inflammatory response syndrome.

**Table 5.** Success Rate of all Three Risk Groups Patients with Different Types of Interventions

	Conservative Management (13)			DJ Stent +/− PCD Placement (40)			PCN +/− PCD Placement (37)		
	Success	Failure	Total	Success	Failure	Total	Success	Failure	Total
Good risk	4 (80%)	1 (20%)	5	10 (90.9%)	1 (9.1%)	11	3 (100%)	0	3
Intermediate risk	2 (25%)	6 (75%)	8	12 (60%)	8 (40%)	20	18 (94.7%)	1 (5.3%)	19
Poor risk	0	0	0	2 (25%)	6 (75%)	8	10 (58.8%)	7 (41.2%)	17

PCD, percutaneous drain; PCN, percutaneous nephrostomy.
